# Parental Support, Beliefs about Mental Illness, and Mental Help-Seeking among Young Adults in Saudi Arabia

**DOI:** 10.3390/ijerph17155615

**Published:** 2020-08-04

**Authors:** Alaa Mahsoon, Loujain Sharif, Maram Banakhar, Nofaa Alasmee, Esraa Almowallad, Razan Jabali, Amjad Bahamil, Sara Assur

**Affiliations:** 1 Nursing Public Health, King Abdulaziz University, Jeddah 21589, Saudi Arabia; Mahsoon@kau.edu.sa (A.M.); ahbbanakher3@kau.edu.sa (M.B.); 2 Nursing Mental Health, King Abdulaziz University, Jeddah 21589, Saudi Arabia; lsharif@kau.edu.sa (L.S.); nalasmee@kau.edu.sa (N.A.); 3 Nursing, King Abdulaziz University, Jeddah 21589, Saudi Arabia; ialmowallad@stu.kau.edu.sa (E.A.); rjabali0001@stu.kau.edu.sa (R.J.); abahameil@stu.kau.edu.sa (A.B.)

**Keywords:** cross-sectional, beliefs, mental help-seeking, mental illness, parental support, young adults

## Abstract

Mental illness is not uncommon among young adults, but negative attitudes towards mental disorders and lack of parental support might be associated with hesitancy in seeking professional help. This study aimed to examine the relationships of parental support, beliefs about mental illness, and mental help-seeking among young adults in Saudi Arabia. This quantitative cross-sectional study included a convenience sample of 236 young adults (ages 18–25) with the majority of the total (86.4%) being female. Data were collected via three self-administered questionnaires: The Perceived Parental Support Scale, Beliefs toward Mental Illness scale, and Mental Help Seeking Attitude Scale. Results indicated that the participants had a moderately negative attitude toward mental illness, a moderately positive attitude toward parental support, and a highly positive attitude toward mental help-seeking. No significant relationships were found among the study variables. The study highlights that despite young adults’ positive attitude toward mental help-seeking and parental support, they have negative views toward people with mental illnesses. Educational programs in schools and media are needed to improve attitudes and enhance readiness to interact with people with mental illness.

## 1. Introduction

Mental illness is a neglected issue in Saudi Arabia, as evidenced by the lack of reported data on the prevalence of mental illness in the general population. Further, negative attitudes toward mental illness among the Saudi population, as well as hesitancy toward seeking mental help, have been reported in the literature [1]. For example, reports indicate that treatments for mental illnesses might be seen as useless, time-wasting, and expensive [1].

People diagnosed with mental illness are often rejected by the community and considered a burden to their families; thus, they, and their relatives, often hide the disorder from others because they fear public disapproval. Various studies have found that most people isolate themselves from individuals with mental illness, considering them dangerous and incapable of friendship [1,2]. Negative attitudes toward mental illness promote public discrimination against and stigmatization of patients with mental disorders and may cause them to withdraw from social interactions, which is likely to aggravate their mental illness condition [3].

Parental support is essential to a child’s recovery from mental illness. Research indicates that children diagnosed with mental disorders thrive in an environment where they are accepted and encouraged [4]. Such situations allow youths to build up their self-confidence, social skills, and emotional control abilities [4]. Parents are commonly involved in the mental help-seeking progression of youths suffering from mental health disorders [5]. In addition, parents have a strong influence on encouraging their children to seek help and finding the right path for managing their children’s psychological problems, and parents who have stable marriages and shared intimacy tend to have better attitudes toward mental health disorders [6].

### 1.1. Significance of the Problem

There is a growing concern about the burden and effect of mental disorders on people’s health, their social lives, and economies worldwide [7]. It was found that among 160 patients who attended primary healthcare centers in the capital of Saudi Arabia during three months in 2017, 28.5% reported cases of mental health disorders [8]. Further, the Saudi National Health & Stress Survey reported that 2 in 5 Saudi youth meet the criteria for having a mental health condition sometime in their life and only 5% of Saudis seek professional treatment for their mental illness [9]. It is important to emphasize that supportive attitudes toward mental disorders are vital to minimizing the stigma around mental illnesses and encouraging people to seek help from the appropriate sources [10]. Nevertheless, a study of 650 Saudi adults (age 18 years and older) revealed that most of the participants (87.5%) indicated a lack of knowledge about mental illness. Moreover, 66.5% of the participants reported negative attitudes toward people with mental illness in relation to successful treatment, maintaining a job, and getting married, as well as toward their obtaining professional help [1]. Thus, it is important to draw attention to attitudes toward mental illness and mental help-seeking among young adults in Saudi Arabia. 

It is also critical to explore attitudes towards parental support. Young adults aged 18 to 25 usually live with their parents in the same house in the culture of Saudi Arabia, which allows parents to be involved in a variety of aspects of their children’s lives, including their psychological problems. Parents’ behaviors and attitudes have a powerful impact on the mental, psychological, and emotional well-being of their children [11]. Parents should be actively involved in their children’s lives by offering support and encouragement when required. Such an approach will help children to manage and defeat mental disorders [4]. However, in 2019, the general divorce rate in Saudi Arabia reached 3.20 divorces per 1000 in the Saudi married population [12]. It was discovered that separation among parents is significantly related to depression, anxiety, and stress among young Saudi females [13].

### 1.2. Literature Review

Negative attitudes toward mental disorders appear to have an adverse impact on patients with mental disorders. Abolfotouh et al. found that 66.5% of people reported negative attitudes toward people with mental illness, as discussed above [1]. Another study conducted with 557 undergraduate college students showed that half of the students (52.5%) held negative attitudes towards persons with mental illness, believing them incapable of friendships [14]. A further study reported that one-third of 3464 Saudi participants had a hesitant attitude toward mental illness and persons with mental illness [15]. However, another recent study discovered that 90% of Saudi adults sampled believed that patients with mental illness deserve respect [16]. Nevertheless, 35.8% of people in the same study believed that patients with mental illness leaned toward dangerous and aggressive behavior, and 33.2% thought that society would be more secure if patients with mental illness were hospitalized [16]. 

Researchers indicate that religion, culture, and urbanization influence parents’ beliefs on psychological disorders. For example, James et al. argue that conservative cultural and religious tendencies in the nation contributed to those beliefs [7]. Studies also indicate that, compared to people living in urban centers, those in rural areas are more tolerant of people with mental disorders [17]. Negative attitudes could also be due to a lack of knowledge about mental disorders. In contrast, individuals who possess high levels of education are more knowledgeable about mental illnesses [3]. 

While there are studies exploring attitudes about mental illness, there are scarce data on attitudes toward mental help-seeking among the Saudi population. A study that aimed to explore the attitudes towards mental health help-seeking in 650 Saudi adults aged > 18 years showed that 54.5% of participants reported negative attitudes to help-seeking behaviors, 40.5% reported neutral attitudes, and only 5% reported positive attitudes [1]. Religious, supernatural, and social beliefs can affect how people perceive causes of mental illness and how to deal with it. It was found that, in Saudi Arabia, people strongly believe in demonic possession and the evil eye, which may lead them to seek unprofessional help or help from religious healers before going to mental health specialists [2]. Therefore, there is a need to investigate the attitude toward seeking help from official psychological and psychiatric specialists among Saudi young adults.

Parental support has been defined as “parental behaviors toward the child, such as praising, encouraging and giving physical affection, which indicate to the child that he or she is accepted and loved” [18] (p.176). Parents’ behaviors and attitudes have a powerful impact on the mental, psychological, and emotional wellbeing of an individual [11]. In adulthood, parents continue to be fundamental supporters in helping young adults with mental illness needs [19]. Divorce and separation among parents are problems that may interfere with the psychological wellbeing of their children. A study was conducted in Riyadh on 327 females, aged 12 to 16 years, who disclosed that the prevalence of parental divorce was 13.3% (*n* = 40) while that of parents living separately but not legally divorced was 10.9% (*n* = 33). Taken together, the prevalence of marital discord (divorced or separated) was 24.6% (*n* = 73 out of *n* = 296). The results revealed that separation among parents was significantly related to depression, anxiety, and stress among young Saudi females [13].

The association of mental help-seeking behaviors with parental support has been borne out by the literature [4,11,20,21]; however, this has not been researched among Saudi young adults. One study showed that when young people had positive past experiences and received social support and encouragement from others, such as parents, they had a more positive attitude than others in this age group toward seeking mental help [20]. In addition, better parent–child relationships were associated with greater mental help-seeking intentions [21]. Another study conducted among 1482 students (mean age 17 years) disclosed that positive parenting had an association with greater intentions of seeking mental help from professional sources; 88% of parents indicated they had influenced their young children to obtain mental health services [4]. Wahlin and Deane examined the mental help-seeking process among 256 young people, reporting that 90% of parents of young people stated that parents are more influential than any other source regarding seeking mental help from professional services [11]. Additionally, one study that examined parental support and mental help-seeking attitude in adolescents showed a small but significant positive relationship between parental support and help-seeking intentions among 1482 Australian students aged 16 to 18 years [4]. Another study exploring parents’ attitudes toward their children’s mental disorders and help-seeking behaviors among 400 Iranian parents revealed a significant relationship between seeking help from official sources and fathers’ higher level of education [6].

Much of the literature indicates that attitudes toward mental illness and mental help-seeking are correlated concepts, and the connection between them seems logical; when young adults live with supportive parents who have accepting attitudes toward mental illness, their attitude toward mental help-seeking will be positive as well [4,6]. However, no studies were found to support, with empirical evidence, the possible existence of the relationships among the variables for young adults in Saudi Arabia. 

In order to better understand and improve the mental health of Saudi young adults, it is crucial to assess their attitudes toward parental support, mental illness, and mental help-seeking. At present, there is little research regarding the role of parenting and parental support with mental health illnesses in Saudi Arabia. Additionally, there is a lack of empirical evidence to support the hypothesis that if parenting support and beliefs about mental illness are positive, attitudes toward mental help-seeking will be positive as well. Therefore, it worth exploring the attitudes of, and possible relationships between, parental support, beliefs about mental illness, and mental help-seeking attitudes among young adults in Saudi Arabia.

### 1.3. Objectives

In order to examine the potential relationships between these attitudes, three specific objectives were developed: (1) to assess the attitudes of young adults about parental support, mental illness and mental help-seeking, (2) to examine the relationships between parental support, beliefs toward mental illness, and mental help-seeking attitude, and (3) to observe the difference in the mean scores of these three measures across the characteristics (age groups, gender, occupation, educational status and parents’ status) of study subjects.

## 2. Materials and Methods 

### 2.1. Research Design

A cross-sectional design was used among young adults (aged 18–25 years) in Saudi Arabia. 

### 2.2. Sampling and Sample Size

Participants were young adults recruited from the general public. Inclusion criteria were (a) young adults aged from 18 to 25 who were (b) able to speak and read English. There were no exclusion criteria for those who met the inclusion criteria.

A convenience sample of 236 young adults was enrolled in this study. The relationships between parental support, beliefs toward mental illness, and mental help-seeking attitudes have not been previously studied; hence, a relevant published effect size is not available. Therefore, the estimation for a medium effect size was considered. The estimated medium effect size is 0.30 for correlations with two-tailed tests at 0.05 level of significance and power of 0.80 [22], so the required sample size for correlation was 84 in this study, as calculated by G*Power 3. 1. 

### 2.3. Measures

The questionnaire in this study was comprised of four measures: demographic data, the Perceived Parental Support (PPS) Scale, the Mental Help Seeking Attitude Scale (MHSAS), and the Beliefs Toward Mental Illness Scale (BMI). 

Demographic data collected included gender, age, country of residence, occupation, educational level, marital status, and parents’ relationship/marriage status.

The Perceived Parental Support (PPS) Scale [23], a valid and reliable tool used with multiple populations, demonstrated a Cronbach’s alpha between 0.77 and 0.87. The PPS consists of a five-item scale to measure parental general support [23]. The PPS starts with a question, “How easy or hard is it for you to receive the following from your parents?” followed by five items (a) “Caring and warmth,” (b) “Discussions about personal affairs,” (c) “Advice about studies,” (d) “Advice about other issues (projects) of yours,” and (e) “Assistance with other things.” The responses are rated on a 4-point Likert scale from *very difficult* (1) to *very easy* (4). The possible score values range from 5 to 20 with higher mean scores indicating greater levels of perceived parental support.

The Mental Help Seeking Attitude Scale (MHSAS) [24] is a 9-item scale designed to measure respondents’ overall evaluation of their attitude toward seeking help from a mental health professional if they were to find themselves dealing with a mental health concern. The resulting mean score can range from a low of 1 to a high of 7. The possible score values range from 9 to 63. A higher score indicates a more positive attitude toward seeking help [24]. The HSAS has demonstrated initial evidence of reliability with a Cronbach’s alpha of 0.93 [24].

The Beliefs Toward Mental Illness Scale (BMI) is a 21-item self-report measure of negative stereotypical views of mental illness [25]. The measure holds promising evidence of validity and reliability with a Cronbach’s alpha of 0.82 [25]. The BMI involves three subscales: “dangerousness,” “poor social and interpersonal skills,” and “incurability.” Items are rated on a six-point Likert scale ranging from *completely disagree* (0) to *completely agree* (5). The possible score values range from 0 to 105. Higher scores indicate greater levels of negative belief toward mental illness. 

### 2.4. Data Collection Procedure

Primary data collection was done using a questionnaire. The participants were approached using online social media platforms including WhatsApp, Facebook, and Twitter. The survey was distributed using an online tool and was composed of 35 items consisting of the demographics, PPS, MHSAS, and BMI. The questionnaire for data collection remained available online from March 2020 to April 2020. 

### 2.5. Data Analysis

Data entry and analysis were performed using the latest version of the Statistical Package for the Social Sciences (SPSS) 26.0 version (IBM Inc., Chicago, IL, USA). Descriptive statistics were used, including frequencies for all study variables. Means and standard deviations were reported for continuous variables, counts, and percentages for categorical variables. For inferential statistics, Pearson’s correlation coefficient was performed to test the significance and the strength of relationships between continuous variables. Student’s t-test and one-way analysis of variance were also used to compare the mean scores of the three instruments in relation to the categorical variables. The reliability of three instruments was assessed by calculating Cronbach’s alpha. A *p*-value of ≤0.05 and 95% confidence intervals were used to report the significance and precision of the results.

### 2.6. Ethical Considerations

Ethical approval for this study was obtained from the Ethics and Research committee at the Nursing Faculty, King Abdulaziz University. Study enrolment was voluntary and data collection was anonymous, as the participants were not asked to include their names. Detailed information about the study was included on the first page in the online survey tool. Hence, before the participant started the survey, they had the chance to read the information provided and decide whether they were interested in being enrolled in the study. Informed consent was implied by the participants completing and submitting the survey.

## 3. Results

### Sample Characteristics

Out of 270 total respondents, 34 were excluded because their age was not within the inclusion criteria. Most subjects (66.5%) were older than 20, and the majority of the total (86.4%) were female. About 84% of study subjects were students of a variety of disciplines, and remaining participants specified whether they were employed or unemployed. More than 50% had a bachelor’s degree, and 96.2% were single. The parent’s status was “together” in 78% of the study subjects; the remaining parents were separated, divorced, or “other” (see Table 1).

The internal consistency of the three measurements was assessed and indicated acceptable reliability; Cronbach’s alpha of all three measurements was above 0.75. Descriptive statistics of the total scores of the three measurements are also given in Table 2.

From the descriptive statistics, it can be observed that the study participants showed a moderately negative belief toward mental illness and indicated a moderately positive attitude toward parental support. Additionally, the participants’ mean scores of mental help-seeking indicate a highly positive attitude toward mental help-seeking. To observe the linear relationship among the three outcome variables (scores of three measurements), we used Pearson’s correlation analysis, which showed no significant relationship among the three variables. 

The comparison of mean scores of three measurements (parental support, beliefs towards mental illness, and metal help-seeking attitude) in relation to the study demographic variables (age groups, gender, occupation, educational status, and parents’ status) showed no statistically significant difference in the mean scores of any of these three measurements across the any of the five study variables. This bivariate comparison does not provide any evidence that the mean scores are statistically significantly different across the categories of the five study variables (see Table 3).

## 4. Discussion

In this study, participants showed a moderately negative attitude toward mental illness and people with mental illnesses overall. Specifically, about 40% of the participants reported a negative attitude toward the capability of a person with mental illness to be functional as a parent, make friends, and be a trustworthy person within a team. Participants’ attitude toward other aspects, such as people whether with mental disorders are likely to be criminals or have unpredictable behaviors [25], was mostly positive or favorable. Thus, the attitude of the participants could be considered mixed according to each item analysis in the BMI tool. The study results are consistent with a study that was conducted with a similarly aged population in Saudi Arabia [14]. Among 575 undergraduate students, it was found that they reported a mixed attitude toward people with mental illness, with 32% reporting that they could not maintain a friendship with a person who has a mental illness and 82.5% reporting that people deserve the same rights as anyone else [14]. Moreover, 66.5% of 650 Saudi adults aged > 18 years old reported negative attitudes toward people with mental illness concerning treatment, work, marriage, and recovery, and toward getting professional help [1]. This finding was incongruent with another study that was done with 232 undergraduate students who indicated positive attitudes towards all aspects of mental illness [26]. Based on the available data, it is evident that Saudi young adults have a varied attitude toward mental illness and people with a mental illness, which was proved by a previous study conducted with 3464 Saudi adults recruited from the public [15]. A possible explanation for our study’s result of a moderate negative attitude toward mental illness can be explained by the low mental health literacy among young adults in Saudi Arabia. A study on mental health literacy among Saudi youth uncovered that 575 Saudi young adults showed intermediate mental health literacy levels, viewing mental illness as God’s punishment, the evil eye, magic, or demonic possession [14]. A similar finding was noted in a qualitative study done in Saudi Arabia that found that mental illnesses among Muslims were perceived as possession by supernatural forces or punishment from God [27]. Moreover, stigma or shame of mental illness is not an uncommon finding among the Saudi community [2,27].

In this study, the results indicated that young adults had a moderately positive attitude toward their parental support. A marked number of separated parents were noted, as 30% of participants reported having divorced parents. This might be a possible explanation for participants’ reporting a moderate rather than high positive attitude toward parental support. A separation or divorce is a highly stressful emotional experience, and long-term negative parental conflict can negatively impact children’s psychological wellbeing. Although no studies have examined the attitude of Saudi young adults about parental support, one study done in Saudi Arabia using Jeeluna^®^ national survey data (12,121 observations) found that a poor relationship with parents was significantly associated with feeling sad or hopeless and worried [27].

There is a scarcity of data on the attitude toward mental help-seeking among the young adult Saudi population. The participants in the current study reported a highly positive attitude toward mental help-seeking. However, conflicting evidence was found in the literature [1,14,28]. Researchers found that 45% of 575 Saudi young adults believed that religious healers could treat people with mental illness [14]. Similarly, it was found that spiritual approaches, such as exorcism, are prominent in the Saudi population [28]. The conflicting evidence might be due to the increasing amount of mental health service in the past few years in Saudi Arabia [29]. Further, people’s attitude could be changing with time because of heightened awareness. Further longitudinal studies with larger samples are needed to clarify the conflicting evidence.

The data revealed no significant relationship between the three variables despite their logical associations. No studies have been done in Saudi Arabia previously for comparison; however, our results are congruent with a study that indicated no significant relationship between beliefs toward mental illness and help-seeking among young adults in the United States [30]. A contradicting result was found in a study that disclosed a small but significant positive relationship between parental support and help-seeking intentions among 1482 Australian students aged 16 to 18 years [4]. Overall, a possible explanation for our study’s finding might be young adults’ need for independence; too much parental involvement with their help-seeking services could feel pressuring or undesirable to young adults. A study conducted with young adults in China showed that those who had higher levels of parental involvement were more likely to withdraw from professional mental health services [31]. Future studies using advanced mixed-method approaches are recommended to reveal more reliable evidence.

No association was found between the outcome variables and the demographic variables of the current study. Overall, it was difficult to link the current study findings with comparable studies, as there is a scarcity of studies examining the relationships of similar variables in the literature. The differences between means in the education variable were near significance (*p* = 0.06). However, this result was congruent with a study that has been done on 650 Saudi adults, which revealed that education level was not correlated with the attitude toward mental illness and mental help seeking. Nevertheless, the same study reported that employment and male gender were positively correlated with the attitude toward help-seeking [1]. It was not surprising that there was no significance detected of gender and employment with the outcome variables due to lack of variability in the current study sample, where the majority of the sample was females and students. Another study conducted among 1482 students disclosed that parental authoritativeness was associated with greater intentions of seeking mental help from professional sources [4]. However, in the current study, the examined variable was parental status whether separated or together, which might not have necessarily indicted a parenting style. Further studies are recommended to examine the correlation between the different parent variables and mental health variables. 

The current study limitations include use of self-reported questionnaires. Self-reported data cannot always be independently verified and present a potential source of bias such as recall bias, where participants tend to seek social desirability, hide errors, and exaggerate their knowledge. In addition, this study is limited because of the use of a convenience sample due to limited time and resources with the majority of the enrolled participants were females. However, in the culture of Saudi Arabia women have been understudied in the past [32]. Hence, including more women to make their voice heard is desirable. The use of a cross-sectional design could be a potential limitation as well, because this design examines variables at a single point in time, while some study variables may change over time. For example, the attitudes of participants would best be captured over time, because feelings are subject to change. Finally, the cross-sectional design may have limited detection of possible relationships among the study variables. A longitudinal design is suggested for future studies looking to reveal more reliable data.

## 5. Conclusions

The perception of young adults toward mental illnesses and people with mental illnesses was moderately negative. This result highlights the possibility of low mental health literacy among this young adult population, which could increase the stigma and negative stereotypes among clients with mental illness. The study showed that young adults positively perceived their parents’ support; however, the divorce rate among the participants could not be ignored. Moreover, the results indicated that young adults would most likely seek professional mental help, evidenced by their highly positive attitude toward mental help-seeking. There was no relationship between parental support, beliefs toward mental illness, and mental help-seeking attitude. It is highly recommended that education about mental health and complications of stigma be encouraged and applied in schools, universities, awareness-raising events, and the media. Finally, making community mental health services easily available might help in reducing stigma and providing early detection and treatment of young adults with psychological problems.

### Recommendations

Based on the study findings, multiple recommendations are suggested. Young adults must be educated about mental illnesses at an early age to improve their understanding of mental illness, decrease negative attitudes and stereotypes against people with mental illness, and guide them to available professional mental help services. For example, it might be useful to implement mental health literacy events in schools and public places, such as shopping malls, and create trusted media platforms to raise awareness surrounding mental illness and acknowledge available resources. Moreover, customized workshops for parents to minimize the negative psychological impact of separation and divorce on their children are recommended. Finally, there is a need to conduct similar studies using mixed-methods and longitudinal approaches and recruit larger sample sizes to enhance the accuracy of the findings and enrich the literature on the progression of the attitudes toward and knowledge about mental illness in Saudi Arabia.

## Figures and Tables

**Table 1:** Distribution of characteristics of study subjects (*n* = 236).

Characteristics	No. (%)
Age Groups (in Years)	
≤20	79(33.5)
>20	157(66.5)
Gender	
Female	204(86.4)
Male	32(13.6)
Occupation	
Student	199(84.3)
Employed	24(10.2)
Unemployed	13(5.5)
Education	
High school	90(38.1)
Bachelor’s degree	132(55.9)
Other	14(5.9)
Marital Status	
Single	227(96.2)
Married	8(3.4)
Divorced	1(0.4)
Parents’ Status	
Together	184(78)
Separated	12(5.1)
Divorced	27(11.4)
Other	13(5.5)

**Table 2:** Reliability (internal consistency) of three instruments and descriptive statistics of outcome variables: Parental Support, Beliefs Toward Mental Illness, and Mental Help-Seeking Attitude (*n* = 236).

Instruments and Outcome Variables	Cronbach’s Alpha (95% CI)	Mean (SD)	Min-Max	Possible Score
Parental Support	0.85 (0.82,0.88)	15.24 (3.97)	5–20	5–20
Beliefs Toward Mental Illness	0.80 (0.76,0.84)	50.37 (14.21)	23–100	0–105
Mental Help-Seeking Attitude	0.88 (0.86,0.90)	45.21 (11.49)	23–63	9–63

**Table 3:** Comparison of mean values of three outcome variables in relation to study demographic variables.

Study Variables	Outcome Variables								
	Parental Support			Beliefs toward Mental Illness			Mental Help-Seeking Attitude		
	Mean (Sd.,)	*t*-Value/*f*-Value	*p*-Value	Mean (Sd.,)	*t*-Value/ *f*-Value	*p*-Value	Mean (Sd.,)	*t*-Value /*f*-Value	*p*-Value
Age Groups									
≤ 20 yrs	14.91 (3.9)	-0.80	0.42	20.73 (6.9)	1.57	0.12	49.89 (13.6)	1.30	0.19
>20 yrs	15.35 (4.0)			19.18 (7.3)			47.40 (13.9)		
Gender									
Female	15.85 (3.8)	1.25	0.21	19.43 (7.2)	−0.29	0.77	47.17 (12.7)	−0.59	0.56
Male	15.04 (4.0)			19.77 (7.1)			48.50 (14.1)		
Occupation									
Student	15.03 (3.9)	1.41	0.24	19.92 (7.1)	1.84	0.16	48.03 (13.9)	0.14	0.87
Employed	15.83 (4.3)			19.88 (7.3)			49.42 (16.0)		
Unemployed	16.69 (3.1)			16.0 (7.5)			49.23 (7.7)		
Education									
High school	14.73 (4.0)	1.02	0.36	20.72 (6.3)	1.53	0.22	47.18 (13.9)	2.81	0.06
Bachelor’s	15.50 (3.9)			19.16 (7.5)			49.54 (14.0)		
Other	15.40 (4.1)			18.10 (8.8)			39.70 (6.6)		
Parents’ Status									
Together	15.32 (3.9)	0.53	0.66	20.10 (7.0)	1.28	0.28	48.15 (13.8)	0.57	0.64
Separated	15.58 (3.5)			17.25 (6.6)			52.67 (14.6)		
Divorced	14.33 (4.2)			19.33 (8.0)			46.48 (14.2)		
Other	15.0 (4.9)			16.92 (8.0)			49.08 (13.5)		
